# Community-based directly observed treatment for TB patients to improve HIV services: a cross-sectional study in a South African province

**DOI:** 10.1186/s12913-018-3074-1

**Published:** 2018-04-07

**Authors:** Embry M. Howell, N. Gladys Kigozi, J. Christo Heunis

**Affiliations:** 1 The Urban Institute, Health Policy Center, 2100 M St., N.W, Washington, D.C., 20037 USA; 2 Centre for Health Systems Research & Development, University of the Free State, P.O. Box 399, Bloemfontein, 9300 South Africa

## Abstract

**Background:**

There is uncertainty about how directly observed treatment (DOT) support for tuberculosis (TB) can be delivered most effectively and how DOT support can simultaneously be used to strengthen human immunodeficiency virus (HIV) prevention and control among TB patients. This study describes how DOT support by community health workers (CHWs) was used in four municipalities in the Free State province – a high TB/HIV burden, poorly-resourced setting – to provide HIV outreach, referrals, and health education for TB patients.

**Methods:**

The study was part of a larger cross-sectional study of HIV counselling and testing (HCT) among 1101 randomly-selected TB patients registered at 40 primary health care (PHC) facilities (clinics and community health centres) across small town/rural and large town/urban settings. Univariate analysis of percentages, chi-square tests and t-tests for difference in means were used to describe differences between the types of TB treatment support and patient characteristics, as well as the types of – and patient satisfaction with – HIV information and referrals received from various types of treatment supporters including home-based DOT supporters, clinic-based DOT supporters or support from family/friends/employers. Multivariate logistic regression was used to predict the likelihood of not having receiving home-based DOT and of never having received HIV counselling. The independent variables include poverty-related health and socio-economic risk factors for poor outcomes. Statistical significance is shown using a 95% confidence interval and a 0.05 *p*-value.

**Results:**

Despite the fact that DOT support for all TB patients was the goal of South African health policy at the time (2012), most TB patients were not receiving formal DOT support. Only 155 (14.1%) were receiving home-based DOT, while 114 (10.4%) received clinic-based DOT. TB patients receiving home-based DOT reported higher rates of HIV counselling than other patients.

**Conclusions:**

Public health providers should train DOT supporters to provide HIV prevention and target DOT to those at greatest risk of HIV, particularly those at greatest socio-economic risk.

## Background

As highlighted in the Sustainable Development Goals (SDGs) [1], the world continues to face high rates of poverty and associated population health challenges, including the joint epidemics of tuberculosis (TB) and human immunodeficiency virus (HIV) [2]. High rates of TB-HIV co-infection necessitate HIV testing of all TB patients, yet, only 55% of notified TB patients in South Africa had a recorded HIV test result in 2015 [3]. With only about half of notified TB patients knowing their HIV status, the shortfalls and deficiencies in integration between TB and HIV services in the country become clear. Lack of progress in successfully integrating traditionally separate TB and HIV services into parallel programmes, staffing and approaches negates patients’ preference for combined services and results in wastage of scarce health resources and patients’ time and finances [4].

Both the link between TB and poverty [5–7] and the link between HIV and poverty [8–10] are well established. The Gini index for income inequality – an indicator for achieving SDG 10 to reduce inequality within and among countries – for South Africa was 63.4 in 2011 [11]. In the same year, 16.6% of the country’s population lived below the poverty headcount threshold of ZAR 13.81/US$ 1.90 (2011 exchange rate) per day [12]. The Free State is one of the poorest of the country’s nine provinces. Just more than 5% of the national public health-sector dependent population – of whom more than 80% are African and thus historically and socioeconomically disadvantaged – live in the Free State [3]. At ZAR 91994.00/US$ 12653.92 (2011 exchange rate), average annual household income in the province in 2010/2011 was substantially lower than that for the country as a whole at ZAR 119542.00/US$ 16443.19 (2011 exchange rate) [13]. In 2016, the Free State also had the lowest life expectancy for both males and females of all the provinces [14].

South Africa is extraordinarily challenged by the TB-HIV co-epidemic. The World Health Organization’s (WHO) global TB report shows that in 2016 the country had the highest total TB incidence rate in the world at 781 cases per 100,000 population compared to 140 cases per 100,000 population globally [15]. Further to the report, the HIV-positive TB mortality rate in South Africa was 181 per 100,000 population compared to five cases per 100,000 population globally and HIV prevalence in incident TB cases was 59% compared to 10% globally. In 2015, the Free State reported 15,833 cases of TB and that, in 2014, 70.3% of TB patients were HIV co-infected [3]. In 2014, the TB death rate in the province was substantially higher than that recorded for the country as a whole. A review of cases in the electronic TB register in the province from 2003 to 2012 revealed that both HIV co-infection and unknown HIV status were independently associated with increased likelihood of mortality while on TB treatment [16].

Directly observed support (DOT) continues to be commended as an efficacious approach to overcome interruption in both drug-susceptible TB (DS-TB) [17] and multidrug-resistant TB (MDR-TB) [18] treatment. Uncertainty however exists in the global and national guidelines about how and by whom DOT support for TB can be delivered most effectively and how DOT can simultaneously be used to strengthen uptake of HIV testing and treatment among TB patients. In 2017, the WHO conditionally recommended that the following treatment administration options may be offered to patients on DS-TB treatment: firstly, community- or home-based DOT was recommended over clinic-based DOT or unsupervised treatment; secondly, DOT administered by trained lay providers was recommended over DOT administered by family members or unsupervised treatment; and, thirdly, video observed treatment (VOT) could replace DOT where the technology was available [17].

The South African National Tuberculosis Management Guidelines released in 2014 state that the TB treatment supporter may be a healthcare worker, or a trained workplace or community health worker (CHW) or whoever patients choose to watch them swallowing the tablets “in a way that is sensitive and supportive to the patient’s needs” [19 p. 56]. Further to the guidelines, DOT services must be organised to suit the patients’ circumstances and must be provided as close to their homes as possible, unless patients live close to a clinic and it is convenient for them to take their treatment at the clinic. The guidelines prioritise the following groups for treatment support: children, elderly or infirm patients; those with a history of interrupting treatment, substance or alcohol abuse or mental illness; those who request a treatment supporter; and those who are homeless or living under poor social conditions. There is however considerable variation in how DOT is implemented, how HIV-related services have been integrated into DOT support, and the extent of cross-training to deliver both programmes’ services in South Africa.

Due to the wide use of an intensive service (directly observing patients taking their pills on a daily basis), DOT has been extensively studied for its impact on retention in treatment and treatment outcomes. Results have been mixed, and controversy remains about whether DOT is effective. Individual studies have shown that integrating DOT with an HIV home care programme improved outcomes for TB patients in Zambia [19]. A study in the Western Cape province of South Africa found community-based DOT to be more cost-effective than clinic-based DOT [20]. A study in Namibia showed that TB patients on the community-based TB treatment option had better cure rates than those on clinic-based DOT or self-administered TB treatment [21]. Another South African study in the Northern Cape province found the effects of DOT to be concentrated among patients undergoing retreatment for TB [22]. Qualitative studies in Ethiopia [23] and Pakistan [24] showed some of the potential advantages for clinics and patients with community-based DOT such as patients not having to travel to a clinic for DOT and alleviation of clinic-based healthcare workers’ time constraints by engaging CHWs.

However, a Cochrane review of 11 clinical trials concluded that TB cure and treatment completion rates were not improved by DOT [25]. A similar conclusion was drawn from a meta-analysis of ten studies (including five trials) which found that DOT was not significantly better than self-administered treatment in preventing microbiologic failure, relapse or adverse drug reactions [26]. Thereupon, a systematic review and meta-analysis of eight studies (including one trial) that compared the effectiveness of community-based versus clinic-based DOT concluded that community-based DOT had higher treatment success, but conceded that the evidence for this was not strong [27]. A systematic review of ten trials and eight quasi-experimental studies found evidence of beneficial effects from DOT with regard to treatment adherence and in terms of cure and treatment success rates, but no beneficial effect in terms of increasing the treatment completion rate [28]. Another systematic review of 23 studies (eight trials) concluded that while there was no convincing evidence that clinic-based DOT was more effective than self-administered treatment, there was convincing evidence that community-based DOT was more effective than self-administered DOT and that community-based DOT was as or more effective than clinic-based DOT [29]. This was corroborated by another systematic review and meta-analysis of eight trials and nine cohort studies finding that community-based DOT did improve TB treatment outcomes [30].

A systematic review and meta-analysis of 31 studies in 22 countries focusing specifically on MDR-TB patients showed that providing DOT for a full course of treatment was associated with a significantly higher treatment success rate [31]. However, this study reported no significant differences in the treatment success rates for DOT variously provided to MDR-TB patients by healthcare providers, family members and private DOT providers or between patients having clinic-based DOT and home-based DOT. Perhaps the most compelling argument in favour of maintaining DOT for both DS-TB and MDR-TB is that no large-scale TB programme without DOT has achieved the global TB targets, while most programmes using DOT achieve or nearly achieve these targets [32]. Nevertheless, it is argued that the effectiveness of DOT still needs to be more rigorously evaluated through a pragmatic experimental trial conducted in real-world programme settings [33].

Research in the Free State province showed relative patient satisfaction with HIV counselling provided to TB patients by both lay counsellors and nurses and recommended that expanded use of lay counsellors in TB/HIV programmes could help mitigate the human resource crisis that had resulted primarily from shortages of nurses [34]. However, despite undergoing a standard 59-day training programme, knowledge deficits among DOT supporters concerning TB, HIV and the link between TB and HIV have been identified [35–37]. Stigmatisation against people living with HIV/AIDS (PLWHA) has also been observed in a survey of CHWs (over half of whom were DOT supporters) in the province observing that about 15% of respondents agreed with the statement “Most people with HIV/AIDS only have themselves to blame” [38].

This study describes how DOT support by CHWs was used in one province of South Africa, the Free State, to provide HIV outreach, referrals, and education for TB patients in 2012. The study addressed the following specific research questions: 1) what type of support did patients receive for their TB treatment (home-based DOT, clinic-based DOT, or support by family/friends/employer?; 2) what were the characteristics of the TB patients who did and did not receive DOT support – in particular, did the most vulnerable patients receive support?; 3) what type of HIV-related services – in particular referrals to HCT – did patients receive from their supporters?; 4) how satisfied were patients with the HIV-related information they received, and how did that vary by type of supporter?; and 5) what effect did DOT support – in particular home-based DOT support – have on receipt of HCT?

## Methods

### Setting and design

The study was part of the cross-sectional baseline study of a larger intervention project to improve uptake of HCT among TB patients in the Free State. A patient survey was conducted in four local municipalities or sub-districts – Matjhabeng, Maluti-a-Phofung, Setsoto and Dihlabeng – in March to April, 2012. These municipalities were chosen purposively, because they are among the most economically deprived in South Africa [39] and because they represent a combination of small town/rural (Setsoto and Dihlabeng) and large town/urban (Matjhabeng and Maluti-a-Phofung) settings. Table 1 shows socio-economic characteristics of the four municipalities in 2011. The study was limited to TB patients who received treatment in primary health care (PHC) facilities (clinics and community health centres), which is the primary source of health care for a majority of South Africans, historically-disadvantaged persons in particular [40]. The research was authorised by the Free State Department of Health, and the Institutional Review Board of the University of the Free State approved the research protocol.

**Table 1:** Characteristics of study areas

Municipality	% Having characteristic			
	Unemployed	Households agricultural	With no schooling	Without piped water inside dwelling
Dihlabeng	28.7%	28.7%	8.9%	56.28%
Maluti-a-Phofung	41.8%	51.6%	8.9%	68.1%
Matjhabeng	37.0%	13.6%	4.6%	45.2%
Setsoto	35.7%	29.1%	8.7%	68.6%

Source: Statistics South Africa, 2011 Census [47–50]

### Sampling and data collection

The estimated sample size – to attain a 15 percentage point effect in uptake of HCT by TB patients with 80% power and two-tailed test significance of *P* < 0.05 – at the end of the intervention period of the overall study – was 295 per municipality, that is 1 180 in total [38]. Within each municipality, ten clinics that served at least ten TB patients a year were randomly selected. Within clinics, a random sample (proportionate to the total clinic patient headcount) of registered TB patients over age 18 was selected to be interviewed. A TB nurse at the clinic informed the sampled patients about the study, obtained their informed consent to participate, and scheduled an appointment for them to come to the clinic for the interview. Patients were compensated ZAR 15.00/US$ 1.82 (2012 exchange rate) for transport to the clinic. Resource constraints did not allow for inflation of the sample size to compensate for respondent attrition, refusal to participate, or non-response to certain question items. The final sample size was 1 101 – 6.7% of the original sample could not be accommodated due to patients not coming to the clinic for their scheduled interview. This was usually because they had either completed treatment or died.

Conducted in either English or Sesotho according to the patient’s preference, the fieldworker-administrated questionnaire schedule took about 45 min to complete. Six questions collected socio-demographic and socio-economic information: 1) sex (male/female); 2) age (continuous); 3) marital/cohabiting status (married/living with a sexual partner or not married/living with a sexual partner); 4) highest formal educational qualification (no/primary school or secondary school/higher); 5) employment status (employed/unemployed); and 6) housing quality (main walling material, kind of toilet facility, and availability of tap drinking water and electricity).

Seven questions inquired about DOT support: 1) who supported the patient while on TB treatment (home-based DOT, clinic-based DOT, support from family/friends/employer, or no one); 2) whether the supporter provided the patient with information on the relationship between TB and HIV/AIDS (yes/no); 3) whether the supporter encouraged the patient to undergo HIV counselling (yes/no); 4) whether the patient ever received HIV counselling (yes/no); 5) whether the supporter was influential in the patient’s decision to undergo HIV counselling (yes/no); 6) how long after the TB diagnosis the patient underwent HIV testing (days); and 7) how the patient rated the DOT supporter in terms of clarity of information about the link between TB and HIV, opportunity to ask questions, thoroughly discussing information about TB and HIV and the benefits of getting tested for HIV, and language used (scale ranging from 1 to 5, where 1 was “very dissatisfied” and 5 was “very satisfied”). Additionally, one question collected clinical information: whether the current episode was the first time the patient had TB (yes/no).

While patients were asked whether they had ever had an HIV test, that response is not part of this analysis. This is because only ten of the 1 101 TB patients reported never having had an HIV test. At the time of the study, it was the protocol for all public health providers to encourage TB patients to have an HIV test and to record the result in the TB register. Results (testing rates) were used as an indicator of health district performance.

### Data analysis

Data were processed and analysed using IBM SPSS statistics for windows, version 24. The significance of difference in proportions was tested using chi-square and t-tests. The multivariate analysis used logistic regression. Regression results are presented as odds ratios with corresponding confidence intervals and *p*-values for statistical significance. The independent variables included in the regression models were poverty-related health and socio-economic risk factors for poor outcomes, including socio-demographic characteristics (gender and age); socio-economic characteristics (marital status, employment status, and housing quality); and clinical status (retreatment for TB). Outcome variables included the following: type of support, type of HIV services received from the supporter, whether the patient received HIV counselling, whether the supporter influenced the patient’s decision to undergo HIV counselling, whether the HIV test took place within one week after referral, whether the supporter influenced the patient’s decision to undergo HIV testing, and the patient’s satisfaction with the supporter’s services.

## Results

### Type of DOT support received

The type of DOT support patients reported receiving is indicated separately by municipality in Table 2.

**Table 2:** Type of support for TB treatment by municipality

Municipality	Number (row %)				Total
	Home-based DOT	Clinic-based DOT	Family/friend/employer support	No one	
Dihlabeng	25 (9.8)	20 (7.9)	76 (29.9)	133 (52.4)	254 (100.0)
Maluti-a-Phofung	58 (22.3)	27 (10.4)	141 (54.2)	34 (13.1)	260 (100.0)
Matjhabeng	30 (10.2)	21 (7.1)	134 (45.4)	110 (37.3)	295 (100.0)
Setsoto	42 (14.4)	46 (15.8)	172 (58.9)	32 (11.0)	292 (100.0)
Total	155 (14.1)	114 (10.4)	523 (47.5)	309 (28.0)	1101 (100.0)

Note 1: Chi-square test for differences in proportions of type of support across municipalities is significant at *P* < 0.001

Note 2: Type of support measured by responses to the question: “Who is supporting you while you are on TB treatment?”

Overall, only 14.1% of patients had DOT support at home, while an additional 10.4% reported DOT support at a clinic. There were significant differences by municipality, with rates ranging from 22.3% home-based DOT in Maluti-a-Phofung to only 9.8% in Dihlabeng. There was also a substantial range for those reporting clinic-based DOT (from 7.1% in Matjhabeng to 15.8% in Setsoto). Almost half of the patients (47.5%) reported support from family/friends/employers with a range from 29.9% (Dihlabeng) to 58.9% (Setsoto). The remainder of the patients stated that they had no support for their TB treatment, fully 52.4% in Dihlabeng.

### Characteristics of patients who received/did not receive DOT support

Table 3 shows several need-related patient characteristics according to the type of support they received while on treatment. Half (50.2%) of patients were male, with no significant gender difference by support type. However, those with DOT support at home were significantly older (mean age 42.0 years) than those supported by family/friends/employers (mean age 38.0 years) and those having no support (mean age 38.3 years).

**Table 3:** Characteristics of TB patients on treatment by type of treatment support received

Characteristic	Number (%) having each characteristic by type of support				Total patients	Significant difference in characteristic by type of support
	Home-based DOT	Clinic-based DOT	Family/friend/employer support	No one		
Total N	155	114	523	309	1101	–
Male (%)	72 (46.5)	69 (60.5)	265 (50.7)	147 (47.6)	553 (50.2)	NS
Mean age (SD)	42.0 (14.3)	40.0 (11.1)	38.0 (12.7)	38.3 (14.3)	38.8 (12.6)	** (Note 2)
Unemployed (%)	125 (80.6)	85 (74.6)	388 (74.2)	225 (72.8)	823 (74.7)	NS
Unmarried and not cohabiting (%)	75 (48.4)	62 (54.4)	290 (55.4)	191 (61.8)	618 (56.1)	*
Very poor housing (%)	22 (14.2)	34 (29.8)	136 (26.0)	62 (20.1)	254 (23.1)	**
No or only primary education (%)	51 (35.5)	41 (36.0)	141 (27.0)	93 (31.1)	326 (29.6)	NS
Undergoing retreatment for TB (%)	25 (16.1)	32 (28.0)	101 (19.3)	66 (21.3)	224 (20.3)	NS

Note 1: *P < 0.05; **P < 0.01; NS = Not significant; Chi-square test for differences in proportions. T-test for difference in means

Note 2: Mean age for DOT at home is significantly different from Family/friends and No one, *P* < 0.01. Other age differences are non-significant

Note 3: Type of support measured by responses to the question: “Who is supporting you while you are on TB treatment?”

Overall, a very large proportion (74.7%) of TB patients were unemployed, but there was no significant difference across their type of support. Among those receiving home-based DOT support, 48.4% were without a partner or spouse at home, compared to 61.8% among those without any support. While it would seem that those without a spouse or partner would be in greater need of support, they were more often without any. About a quarter (23.1%) of TB patients in the sample lived in poor quality housing. These individuals, potentially the highest need group, also were significantly less likely to have DOT support at home. There was no significant difference in level of education by type of support, with between 27.1% and 36.6% of patients having primary school or no education, regardless of their type of support. Finally, national guidelines suggest that those undergoing retreatment for TB should be targeted for DOT support to prevent relapse, but that was not the case. About a fifth (20.3%) reported undergoing retreatment for TB, with no significant difference in that percentage across type of support.

The results from a multivariate logistic regression analysis predicting *not* having DOT support at home are shown in Table 4. Odds ratios are shown for each of the risk characteristics. The multivariate analysis confirmed the results reported above. Older patients were significantly *less* likely to have no support compared with their younger counterparts, while those who were without a partner and those in poor quality housing were *more* likely to have no DOT support at home. While the odds ratios for males, those with only primary or no school education, and those undergoing retreatment for TB were all positive (suggesting they may have also have been more likely to have no home-based DOT support), these odds ratios are not statistically significant. Still, given that these are risk factors that would suggest they should have been targeted for home-based DOT support (especially those undergoing retreatment for TB), it is clear they were not.

**Table 4:** Logistic regression predicting likelihood of not having DOT at home

Control variable	Odds ratio	Confidence interval	Significance
Sex			
Female	1.00	–	NS
Male	1.28	(0.89,1.83)	
Age (continuous)	0.98	(0.96, 0.99)	**
Employment			
Employed	1.00	–	NS
Unemployed	0.65	(0.42, 1.01)	
Marital status			
Married or cohabiting	1.00	–	*
Unmarried and not cohabiting	1.47	(1.02, 2.11)	
Housing quality			
Not in very poor housing	1.00	–	**
In very poor housing	1.84	(1.13, 2.99)	
Education			
Secondary school or higher	1.00	–	NS
No or only primary school	1.15	(0.74, 1.79)	
Undergoing retreatment for TB			
No	1.00	–	NS
Yes	1.42	(0.90, 2.27)	

Note 1: *P < 0.05; *P < 0.01; NS = Not significant

Note 2: Includes all patients in the sample, *N* = 1101

Note 3: Computed using SPSS procedure Binary Logistic

### HIV-related services

Surveyed patients were asked what type of HIV-related services they received from their supporter, both formal DOT supporters and other supporters. These results are shown in Table 5. The percentage of patients with some HIV services from their supporter was high, about two-thirds or more of patients depending on the service and type of support. For example, over 60% of all three groups reported that their supporter influenced their decision to have HIV counselling. The pattern is clear that DOT supporters (either at home or at a clinic) provided significantly more HIV-related services than those being supported by family/friends/employers. With one exception (having an HIV test within one week of TB diagnosis), all the differences in the six HIV-service related responses were statistically significant, and in all cases those with DOT support at home had the largest percentage answering “yes.”

**Table 5:** Types of HIV information and referral received from DOT supporter and patient satisfaction by type of DOT supporter

Outcome	Home-based DOT	Clinic-based DOT	Family/friend/employer support	Significance
	Number (%) answering yes			
Total N	155	114	523	
Information on HIV-TB link from supporter	112 (83.2)	95 (83.3)	435 (72.3)	**
Encouragement for HIV counselling	117 (75.4)	78 (68.4)	323 (61.8)	**
Receipt of HIV counselling	147 (94.8)	106 (93.0)	459 (87.8)	*
Supporter influenced HIV counselling	117 (75.4)	78 (68.4)	322 (61.6)	**
HIV test within week of referral	285 (86.0)	72 (88.9)	92 (83.3)	NS
Supporter influenced HIV testing	117 (75.5)	78 (68.4)	323 (61.8)	**
Satisfaction with supporter for:	Mean			
- Clarity of info about HIV and TB	4.57	4.40	4.42	NS
- Opportunity to ask questions	4.60	4.29	4.39	NS
- Info on HIV and TB was thorough	4.60	4.51	4.47	NS
- Info about the benefit of HIV testing	4.52	4.46	4.47	NS
- Language used	4.71	4.72	4.67	NS

Note 1: *P < 0.05; ** *P* < 0.01; NS = Not significant. T-test for difference in means

Note 2: Among those reporting any support for treatment, *N* = 792

Note 3: Patients rated their satisfaction on a Likert scale ranging from 1 to 5, where 1 was “very dissatisfied” and 5 was “very satisfied”

### Patient satisfaction with DOT supporter HIV-related services

Patients were asked five questions about their satisfaction with the HIV information and referrals received from their supporter. The pattern concerning satisfaction with the services received is not as clear as with receipt of services, in terms of differences between those receiving home-based or clinic-based DOT or receiving support from others. Indeed, there were no statistically significant differences between the groups in any of the satisfaction measures. For example, patients receiving DOT support at home rated their satisfaction with opportunity to ask questions at a mean of 4.6 on a Likert scale ranging from 1 to 5, where 1 was “very dissatisfied” and 5 was “very satisfied.” Patients receiving DOT support at a clinic and those supported by others rated their satisfaction at a mean of 4.3 and 4.4 respectively. These apparent differences were not statistically significant. In terms of satisfaction with the clarity and thoroughness of information on HIV and TB received, information about the benefits of HIV testing, and language used the responses were almost identical across the groups.

### Effect of DOT at home on receipt of HIV counselling

Table 6 shows the results from a logistic regression analysis of the likelihood of *not* receiving HIV counselling, controlling for demographic risk factors and by whether the patient received DOT support at home. Those *without* DOT support at home were almost three times as likely to not have received HIV counselling as other TB patients. Other risk factors for *not* receiving HIV counselling were not significant, except for age. In this case, older age was a risk factor after controlling for DOT support at home and other risk factors.

**Table 6:** Logistic regression predicting likelihood of never having received HIV counselling

Control variable	Odds ratio	Confidence interval	Significance
DOT support			
Home-based DOT support	1.00	–	
No home-based DOT support	2.87	(1.36, 6.06)	**
Sex			
Female	1.00	–	
Male	1.01	(0.69, 1.49)	NS
Age (continuous)	1.02	(1.01, 1.04)	**
Employment			
Employed	1.00	–	
Unemployed	0.80	(0.53, 1.22)	NS
Marital status			
Married or cohabiting	1.00	–	
Unmarried and not cohabiting	0.87	(0.59, 1.28)	NS
Housing quality			
Not in very poor housing	1.00	–	
In very poor housing	0.97	(0.62, 1.54)	NS
Education			
Secondary school or higher	1.00	–	
No or only primary school	0.86	(0.54, 1.39)	NS
Undergoing retreatment for TB			
No	1.00	–	
Yes	1.06	(0.67, 1.67)	NS

Note 1: **P < 0.01; NS = Not significant

Note 2: Includes all patients in the sample, *N* = 1101

Note 3: Computed using SPSS procedure Binary Logistic

## Discussion

The WHO End TB Strategy envisions a TB-free world. The first of the three pillars of the strategy is “integrated, patient-centred care and prevention”; the second is “bold policies and supportive systems”; and the third is “intensified research and innovation” ([41] p.2). “Social protection, poverty alleviation and actions on other determinants of TB” is a component of the second pillar ([41] p.2). One of the ways to do this is by providing effective community-based DOT and HIV services to TB patients at greatest socio-economic risk.

Guidelines for HIV providers in South Africa at the time of the survey (2012) suggested that lay counsellors should play a role in supporting patients [42]. For TB patients, lay counsellors were generally DOT supporters, many of whom had been cross-trained to provide HIV services. While much, but not all, prior research suggests that DOT can be effective for improving TB [22, 28–30] and MDR-TB [31] patient outcomes, research on the effect of DOT specifically on receipt of HIV services is limited. Our research from four municipalities in the Free State province suggests that DOT support – when it was received - facilitated access to a range of HIV counselling services. For example, DOT supporters were encouraging TB patients to take up HCT and educating them about the relationship between TB and HIV. However, at the time of the study the large majority of TB patients in these municipalities were not receiving any formal DOT support. Only 14.1% were receiving home-based DOT, while another 10.4% were receiving clinic-based DOT. This is in spite of the fact that DOT support for all TB patients was the goal of South African health policy at the time.

Prior research [43] and South African guidelines [44] emphasise that DOT support should especially be targeted to the most vulnerable patients. We examined specifically the effect of home-based DOT support and whether it was being targeted to the most vulnerable TB patients. With the exception of older patients being more likely to receive DOT at home, other vulnerable groups were not apparently targeted. Indeed, those in the poorest quality housing were less likely to have home-based DOT support than other TB patients. This finding is similar to that of a recent study in India showing that the most vulnerable TB patients faced the most difficulties in accessing DOT and completing treatment [45].

The advantage of age in receipt of home-based DOT did however not translate into higher rates of receipt of HIV counselling for older patients. It could be that DOT supporters were unassuming or demure about providing HIV-related advice to an older person or may have thought that an older person was unlikely to be in need of HIV counselling, especially when they were a friend or family member.

The study provides important information that is relevant to development of new policies concerning use of CHWs in addressing the TB/HIV co-epidemic. In particular, if cross-training in HIV services can become a standard part of DOT training, CHWs who have frequent contact with TB patients could become an important cadre of front-line HIV prevention personnel in non-clinical community-based settings. Further, in choosing who might receive DOT, it is critical that such a programme target those at the highest socio-economic and clinical risk.

The study is limited to four municipalities in Free State, South Africa at one point in time. In addition, data are self-reported. This particularly may affect how TB patients reports their DOT support. While the term “DOT supporter” implies Directly Observed Treatment, it is possible that the support that patients reported was not necessarily someone observing them taking medication regularly, but was rather someone they saw more occasionally who provided counselling and support. The data do not indicate some important aspects of the HIV services that the DOT supporters, or other supporters, were providing. For example, it is likely that some supporters provided HIV counselling (a direct service), and others encouraged patients to receive counselling at a clinic (a referral). The data do not distinguish these service differences. Another limitation of this analysis is that we did not compare patient treatment outcomes (for example mortality) between patients on DOT and those who were not, since all patients were alive and on treatment at the time of data collection.

## Conclusion

Recent policy developments in South Africa provide some opportunities to improve services for TB patients, through a new “PHC re-engineering strategy,” which includes the deployment of facility-linked outreach and family health teams [46]. Currently, the scope of work of the CHWs serving on these teams, other than in exceptional cases, does not include DOT support. In addition, the teams do not visit households on a daily basis. It is not clear to what extent workers will be cross-trained in both TB and HIV prevention and treatment. Consequently, some of the benefits that co-infected patients might receive from dedicated daily DOT support may no longer be available. It will be important for the national and provincial governments to assure that the benefits of DOT for HIV counselling and prevention – as documented in this study – are not lost with the implementation of the new PHC strategy.

## Abbreviations

CCG: Community caregiver
CHW: Community health worker
DOT: Directly observed treatment
DS-TB: Drug-susceptible TB
HCT: HIV counselling and testing
HIV: Human immunodeficiency virus
MDR-TB: Multidrug-resistant tuberculosis
PHC: Primary Health Care
SDG: Sustainable development goal
TB: Tuberculosis
VOT: Video observed treatment
WHO: World Health Organization
